# Effects of a Ceramide‐Containing Glycinate‐Based Cleanser on the Condition of Oily Skin and Skin Post‐IPL Treatments

**DOI:** 10.1111/jocd.70350

**Published:** 2025-07-17

**Authors:** Yunqiu Xia, Yansi Lyu, Jiahong Yang, Xiaofeng He, Yi Yi, Jingjing Liu, Zailing Zhu, Zheng Du, Yuchao Chen, Yunfei Ai

**Affiliations:** ^1^ Department of Dermatology Children's Hospital of Chongqing Medical University Chongqing China; ^2^ Department of Dermatology Shenzhen University General Hospital Shenzhen China; ^3^ L'Oréal Dermatological Beauty China CeraVe Shanghai China; ^4^ L'Oréal China Research & Innovation Shanghai China

**Keywords:** ceramides, cleansers, skin function

## Abstract

**Background:**

Currently, many studies are exploring the effects of cleansers containing petrolatum and fatty acids on skin condition, but no research has yet investigated the impact of cleansers containing ceramides on skin condition.

**Aims:**

Therefore, this study aims to explore whether a ceramide‐containing glycinate‐based cleanser can improve the skin conditions of oily skin and skin post–intense pulsed light (IPL) treatments, with a particular focus on the skin barrier.

**Methods:**

In this study, 88 volunteers were recruited and divided into two groups. Group 1 (*n* = 44, oily skin) used ceramide‐containing cleansers for 28 days, with subsequent assessment of skin hydration, TEWL (transepidermal water loss), sebum content, and pore count. Group 2 (*n* = 44) received IPL treatment prior to using the same cleansers, followed by evaluation of redness and self‐reported symptoms.

**Results:**

The experimental results demonstrated that in Group 1, after 28 days of product application, skin hydration increased by 47.37%, TEWL decreased by 13.42%, pore number was reduced by 6.92%, and sebum content showed a 79.18% reduction. For Group 2, comparative analysis revealed that following the use of ceramide‐containing cleansers after IPL treatment, skin redness significantly decreased by 14.62% compared to post‐IPL measurements, with concurrent notable improvements in all self‐reported symptoms, including itching and tightness.

**Conclusions:**

Overall, a ceramide‐containing glycinate‐based cleanser can effectively control sebum secretion, reduce pore size, and repair the skin barrier in oily skin. Additionally, it can help reduce redness in IPL‐treated skin. For dermatologists and consumers, this formulation represents a promising option for people undergoing IPL procedures, combining cleansing with barrier repair to minimize post‐treatment downtime.

## 1 Introduction

Cleansers work by eliminating dirt, sweat, sebum, and oils from the skin. This is accomplished through the action of surfactants, which help lift away dirt and dissolve oily substances. Beyond removing impurities, the cleansing process also supports natural exfoliation, contributing to skin rejuvenation. However, the interaction among the surfactants in cleansers, the skin proteins, and skin lipids can be harmful to the skin, especially for skin that has undergone intense pulsed light (IPL) treatment [1]. IPL can improve the appearance of the skin, but it can also cause damage to the skin barrier. Currently, there are no cleansers specifically designed for skin treated with IPL [2].

To achieve skin‐care benefits from cleansers, two key requirements must be met. First, the formulation should minimize surfactant‐induced damage to cutaneous proteins and lipids, as different surfactant classes interact with these skin components with varying affinity and consequently exhibit distinct levels of barrier disruption. Second, the cleanser must effectively deliver beneficial actives (e.g., occlusives, physiological lipids, and humectants) during the washing process to enhance skin hydration while improving both biomechanical properties and visual appearance. Mildness enhancers and moisturizing ingredients, including lipids, occlusives, and humectants, help reduce harmful interactions between surfactants and the skin's proteins and lipids, thereby minimizing skin damage [3]. Moreover, these agents have a restorative effect, replenishing the skin lipids that are lost during washing.

At present, numerous studies are examining the effects of cleansers with petrolatum and fatty acids on skin health [4, 5]. While extensive research has investigated the combined effects of ceramide‐containing cleansers and moisturizers on skin [6], the specific impact of stand‐alone ceramide‐based cleansers remains unexplored in the current literature. Ceramide is one of the most important physiological lipids that affect the skin barrier. Therefore, this study aims to explore whether a ceramide‐containing glycinate‐based cleanser can improve skin conditions of oily skin and skin post‐IPL treatments, with a particular focus on the skin barrier.

## 2 Materials and Methods

### 2.1 Test Products

The studies are controlled before‐after studies. The studies were conducted using a cleanser formulated with sodium cocoyl glycinate as the surfactant, which also contained a blend of three essential ceramides, namely ceramide 1, 3, and 6‐II (ceramide EOP, ceramide NP, and ceramide AP). The participant selection focused exclusively on female subjects because IPL treatments are predominantly requested by women in the Chinese market, as reflected in clinical practice patterns. This gender‐specific recruitment approach ensured the study's relevance to the primary target population.

### 2.2 Clinical Study 1

44 Chinese women were enrolled and took part in this 28‐day study. Volunteers, who fulfill the following criteria of inclusion and exclusion, take part in the study. Inclusion criteria: Women, aged 18–60 years; Forehead sebum level > 100 μg/cm^2^ (measured by Sebumeter) [7]; random cheek stratum corneum moisture content < 60 a.u [8]; random cheek TEWL rate > 15 g/h/m^2^ [9]; at least 30 participants with self‐perceived sensitive skin. Exclusion criteria: (i) intending to get pregnant, pregnant, lactating, or within 6 months of delivery; (ii) those who have known allergies against skin care products or topical medicines; (iii) subjects with dermatological problems or having received any skin or dermatological medical procedures in the test area 3 months before the start of the study.

The product was used twice daily, in the morning and evening, for 28 consecutive days. The measurement time points included before product use (D0), 15 min after the first use (D0 Timm), 4 h after the first use (D0 T4H), 7 days after use (D7), 14 days after use (D14), and 28 days after use (D28). At each time point, the skin moisture content in the stratum corneum was measured using the Corneometer CM825, while the TEWL was assessed with the Tewameter TM Hex. Facial images were captured using the VISIA7 system, combined with IPP for skin pore analysis. Additionally, facial images and skin oil analysis were conducted using the Visioscan VC20 plus and Sebufix 16, with evaluations from dermatologists. All measurements were performed by blinded assessors.

### 2.3 Clinical Study 2

Forty‐four Chinese women were enrolled and took part in this 28‐day study. Volunteers, who fulfill the following criteria of inclusion and exclusion, take part in the study. Inclusion criteria: healthy women, aged 25–55; willing to undergo their first IPL treatment; and willing to participate in the study after the IPL procedure. Exclusion criteria: (i) intending to get pregnant, pregnant, lactating, or within 6 months of delivery; (ii) those who have known allergies against skin care products or topical medicine; (iii) subjects with dermatological problems or having received any skin or dermatological medical procedures in the test area 3 months before the start of the study.

Subjects who underwent IPL treatment on their face used the test product as instructed for 28 consecutive days, applying it twice daily, in the morning and evening. The measurement time points include D0 (before IPL treatment), D0 Baseline (after IPL treatment and before product use), D1 (1 day after product use), D7 (7 days after product use), and D28 (28 days after product use). At each measurement time point, VISIA‐CR was used for facial image collection, and the photos were analyzed for red areas using IPP. At the same time, subjects conducted self‐assessments, evaluating dimensions such as itching, heat sensation, burning sensation, tightness, and stinging. The rating scale ranged from 0 to 9, with 0 indicating “very good,” 4–5 indicating “moderate,” and 9 indicating “very poor.” All measurements were performed by blinded assessors.

### 2.4 Biostatistics and Data Management

The data were analyzed using SPSS 28.0. A normality test was conducted on the data. If the data followed a normal distribution, paired *t*‐tests or independent *t*‐tests were used for statistical analysis. If the data did not follow a normal distribution, the rank‐sum test was applied for statistical analysis. For ordinal data, the rank‐sum test was also used. Missing values due to missed visits were addressed using last observation carried forward (LOCF). Data were analyzed by intention‐to‐treat (ITT), including all randomized participants regardless of protocol adherence, to reflect real‐world effectiveness. The significance level for statistical methods was set at *p* < 0.05.

## 3 Results

### 3.1 Clinical Study 1

**FIGURE 1:**
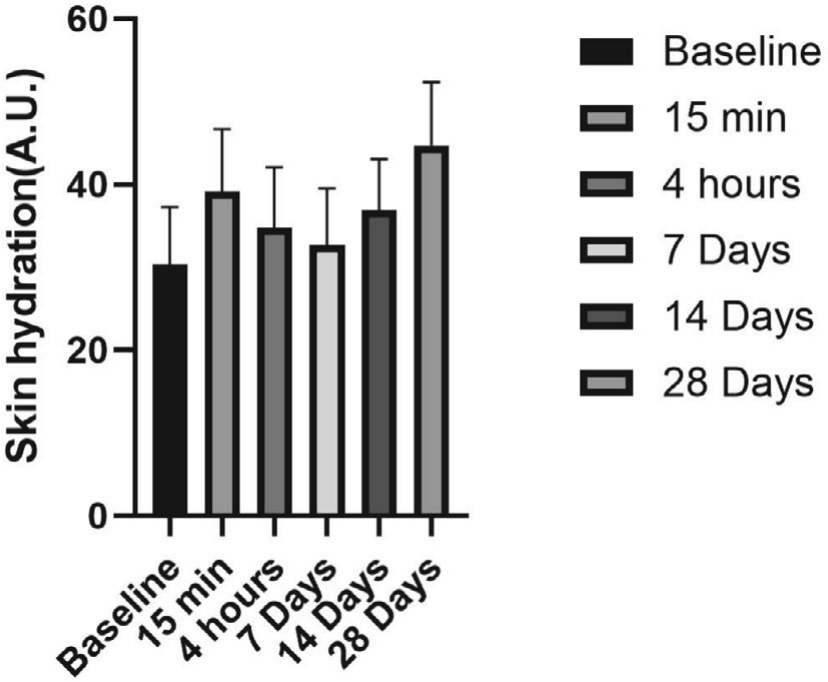
Skin hydration after product use.

The ceramide‐containing cleanser demonstrated a time‐dependent improvement in skin hydration (Figure 1). Immediately following the first application (15 min post‐use), skin moisture levels showed a rapid 28.78% increase (*p* < 0.05), suggesting the formulation's humectant properties provide instant hydration benefits. The most significant enhancement (47.37% increase from baseline) was observed after 28 days of continuous use, indicating that long‐term application allows for cumulative barrier repair and moisture retention effects through ceramide‐mediated mechanisms.

**FIGURE 2:**
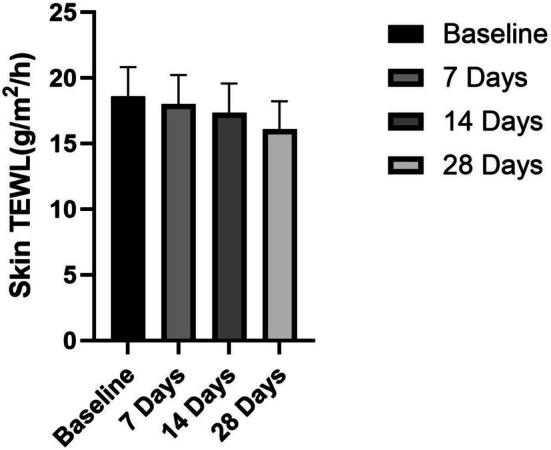
Skin TEWL after product use.

The ceramide‐containing cleanser demonstrated progressive enhancement of skin barrier function, as evidenced by significant reductions in TEWL over time (Figure 2). Initial measurements after 7 days of use showed a modest but statistically significant 3.18% decrease in TEWL (*p* < 0.05), suggesting early‐stage barrier repair. This protective effect became more pronounced with continued use, reaching a 6.69% reduction by Day 14. The most substantial improvement was observed at the 28‐day endpoint, with TEWL decreasing by 13.42% from baseline.

**FIGURE 3:**
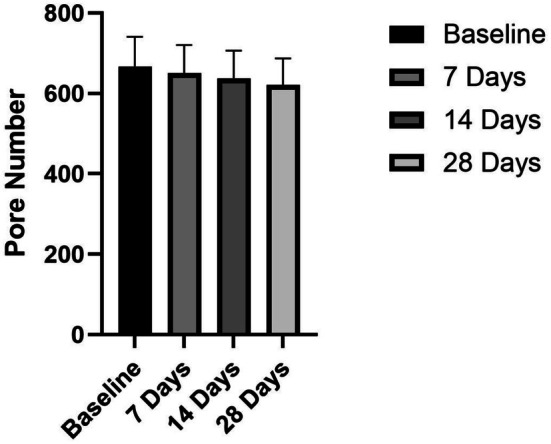
Skin pore number after product use.

Compared to before product use, the number of pores in the cheek area decreased significantly (*p* < 0.05) by 2.53% after 7 days, 4.41% after 14 days, and 6.92% after 28 days (Figure 3).

The ceramide‐containing cleanser demonstrated progressive improvements in pore number over the 28‐day study period (Figure 3). After 1 week of use, participants already showed a statistically significant 2.53% reduction in pore count (*p* < 0.05), suggesting early benefits in sebum regulation. This improvement became more pronounced with continued use, reaching a 4.41% reduction by Day 14. The most substantial effects were observed at the study endpoint, with a 6.92% decrease in pore count after 28 days of regular application.

**FIGURE 4:**
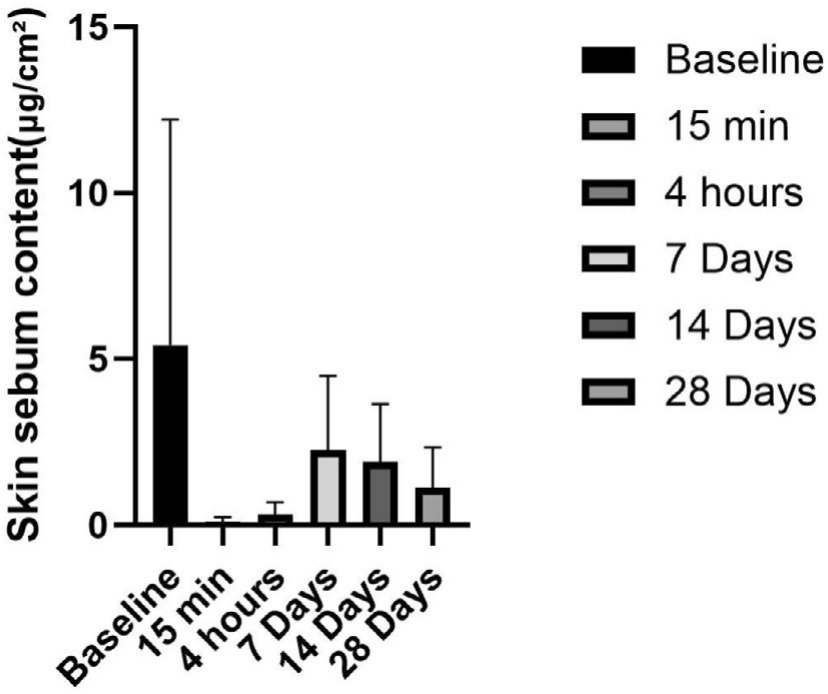
Skin sebum content after product use.

The ceramide‐containing cleanser demonstrated remarkable sebum‐control properties with both immediate and sustained effects (Figure 4). The formulation showed exceptional cleansing efficacy immediately after first use, reducing sebum secretion by 98.01% within just 15 min (*p* < 0.05).

Interestingly, after 1 week of regular use, sebum production showed a 58.53% reduction from baseline, suggesting the product's effects were transitioning from immediate cleansing to longer‐term sebum regulation. By Day 14, the sebum control efficacy improved further to 65.05%, demonstrating progressive benefits with continued use. The most balanced and sustained effect was observed at the 28‐day endpoint, with a 79.18% reduction in sebum secretion.

### 3.2 Clinical Study 2

**FIGURE 5:**
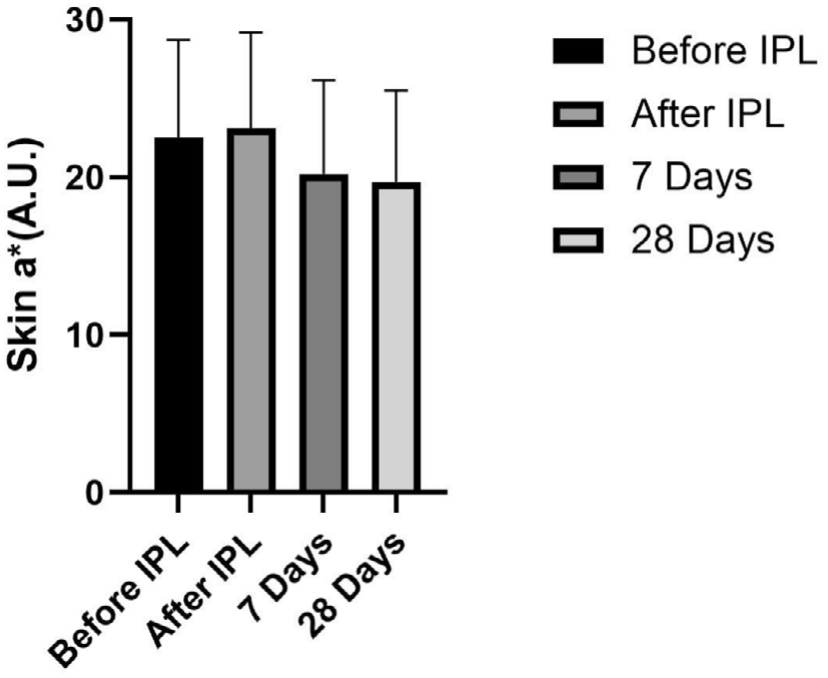
Skin a* values at different time points.

The ceramide‐containing cleanser demonstrated clinically meaningful improvements in post‐IPL erythema, as measured by reductions in a* values (Figure 5). When compared to pre‐IPL baseline measurements, the cleanser achieved a 10.12% reduction in redness after 7 days of use (*p* < 0.05), with further improvement to 12.37% by Day 28. More strikingly, when compared to the immediate post‐IPL measurements (before product use), the redness reduction was even more pronounced—showing a 12.43% decrease after 7 days and a 14.62% improvement after 28 days (*p* < 0.05).

As shown in the table (Table 1), compared to before IPL, the consumer self‐evaluation mean scores for itching, tingling, burning sensation, and tightness significantly (*p* < 0.05) decreased at all time points.

**TABLE 1:** Subject's self‐evaluation results (compared to before IPL).

Evaluation item	Mean score				Change rate (%)			Statistical significance compared to before IPL		
	Before IPL	D1	D7	D28	D1	D7	D28	D1	D7	D28
Itching	2.73	2.52	2.14	1.73	−7.50	−21.61	−36.67	0.012	< 0.001	< 0.001
Tingling	2.66	1.84	1.61	1.30	−30.77	−39.32	−51.28	0	< 0.001	< 0.001
Burning sensation	2.64	1.91	1.73	1.66	−27.59	−34.48	−37.07	0	< 0.001	< 0.001
Tightness	2.89	1.91	1.70	1.27	−33.86	−40.94	−55.91	0	< 0.001	< 0.001

As shown in the table (Table 2), compared to after IPL, the consumer self‐evaluation mean scores for itching, tingling, burning sensation, and tightness significantly (*p* < 0.05) decreased at all time points.

**TABLE 2:** Subject's self‐evaluation results (compared to after IPL).

Evaluation item	Mean score				Change rate (%)			Statistical significance compared to after IPL		
	After IPL	D1	D7	D28	D1	D7	D28	D1	D7	D28
Itching	2.84	2.52	2.14	1.73	−11.20	−24.80	−39.20	< 0.001	< 0.001	< 0.001
Tingling	2.82	1.84	1.61	1.30	−34.68	−42.74	−54.03	< 0.001	< 0.001	< 0.001
Burning sensation	2.66	1.91	1.73	1.66	−28.21	−35.04	−37.61	< 0.001	< 0.001	< 0.001
Tightness	2.95	1.91	1.70	1.27	−35.38	−42.31	−56.92	< 0.001	< 0.001	< 0.001

Overall, the ceramide‐containing glycinate cleanser significantly improved skin hydration, barrier function (TEWL), and sebum control in oily skin, while for post‐IPL, the cleanser reduced redness and alleviated self‐reported symptoms (Table 3).

**TABLE 3:** Summary of key findings.

Parameter	Group 1 (Oily skin, *n* = 44)	Group 2 (Post‐IPL skin, *n* = 44)
Skin hydration	Increased by 47.37% after 28 days (*p* < 0.05)	Not measured
TEWL	Decreased by 13.42% after 28 days (*p* < 0.05)	Not measured
Pore number	Reduced by 6.92% after 28 days (*p* < 0.05)	Not measured
Sebum content	Reduced by 79.18% after 28 days (*p* < 0.05)	Not measured
Redness (*a* values)*	Not measured	Reduced by 14.62% after 28 days (vs. post‐IPL baseline, *p* < 0.05)
Self‐reported symptoms	Not measured	Significant improvement in itching, tightness, burning sensation (*p* < 0.05) at all time points

## 4 Discussion

Skin cleansing is a crucial and well‐established component of treatment for various dermatological conditions [10]. Facial cleansing is essential for eliminating dirt, sebum, and impurities, whether for oily skin or skin treated with IPL. Oily skin (seborrhea) is a frequent cosmetic issue caused by enlarged sebaceous glands producing excessive sebum, resulting in a shiny and greasy appearance. Excess sebum can oxidize into peroxides, irritating the skin and damaging the skin barrier [11]. IPL treatment can damage the skin barrier, triggering pathological and physiological processes like oxidative stress and inflammation. If the skin is not promptly cleansed and the barrier repaired, external irritants will more easily penetrate the skin.

Sebum control is one of the key indicators for measuring a cleanser's effectiveness [12]. This study demonstrates that a ceramide‐containing glycinate‐based cleanser can effectively control skin sebum secretion in both the short and long term, primarily due to the glycinate's excellent oil‐cleansing properties [13]. This finding is consistent with previous research, which demonstrated that cleansers containing amino acid surfactants can reduce sebum production with prolonged use [14]. Furthermore, the study shows that the ceramide‐containing glycinate‐based cleanser can significantly reduce the number of skin pores, which is mainly attributed to the positive correlation between sebum secretion levels and facial pores [15].

The impact on the skin barrier and the skin's erythema response are important indicators of a cleanser's gentleness [16]. This study demonstrates that a ceramide‐containing glycinate‐based cleanser can significantly improve the skin barrier indicator TEWL. This contrasts with previous findings, which indicated that skin TEWL increased after using cleanser [17]. The inconsistency is partly due to the added glycinate, which, according to previous research, is not only gentler than SLES [18] but also less irritating than glutamate‐based surfactants [19]. On the other hand, this is because of the use of biphasic crystallization technology in the product, which involves a dual‐phase liquid crystal system where the sheet‐like lamellar phase and onion‐like lamellar phase coexist. The former generates a rich foam, while the latter enables quick foaming. The coexistence of these two phases allows for the creation of a rich, delicate foam. Previous studies have shown that a rich and delicate foam not only provides consumers with a pleasant sensory experience but also reduces friction against the skin and limits surfactant penetration into the skin, thereby reducing dryness or irritation from cleansing [20]. Furthermore, the inclusion of ceramide NP, ceramide AP, and ceramide EOP also contributes to the formula's effectiveness. Previous studies have shown that ceramides play a critical role in preventing moisture loss during cleansing with surfactants [21] and are highly effective in repairing damaged skin barriers. Specifically, ceramide NP is the most prevalent ceramide found in the human skin's stratum corneum. Additionally, this study reveals that a ceramide‐containing glycinate‐based cleanser can significantly reduce skin redness after IPL treatment. This effect is primarily due to the product's ceramide content, as previous in vitro studies have shown that ceramides have reparative properties for the skin barrier and can lower the expression of inflammatory enzymes and cytokines, thereby providing anti‐inflammatory benefits.

Our study has several limitations. First, the lack of a control group prevents direct comparison with untreated skin, which could help better isolate the specific effects of our ceramide‐containing glycinate‐based cleanser. Second, the relatively short 28‐day study duration may not fully capture the long‐term effects and potential rebound phenomena after discontinuing use. Third, while we measured various physiological skin parameters, the absence of objective skin lipid measurements, such as quantitative ceramide profiling, limits our ability to fully understand the mechanistic basis for the observed improvements in skin barrier function. Additionally, the need to increase the sample size remains to strengthen the statistical significance of the results. Furthermore, the exclusion of male participants may limit extrapolation of findings to male populations, particularly given known gender differences in skin physiology and sebum production. In future studies, integrating skin lipidomics, extending the observation period, including appropriate control groups, and expanding recruitment to include both genders could provide a more comprehensive understanding of the effects of this cleanser formulation.

## 5 Conclusion

Overall, a ceramide‐containing glycinate‐based cleanser can effectively control sebum secretion, reduce pore size, and repair the skin barrier in oily skin. Additionally, it can help reduce redness in IPL‐treated skin. For dermatologists and consumers, this formulation represents a promising option for people undergoing IPL procedures, combining cleansing with barrier repair to minimize post‐treatment downtime.

## Author Contributions

Yunqiu Xia: Conceptualization; writing – preparation of first draft. Yansi Lyu: methodology. Jiahong Yang: software. Xiaofeng He: data management. Yi Yi: supervision. Jingjing Liu: project management. Zailing Zhu: methodology. Zheng Du: data curation. Yuchao Chen: visualization. Yunfei Ai: writing – review and editing. All authors have read and approved the final manuscript.

## Conflicts of Interest

The authors declare no conflicts of interest.

## Data Availability

The data that support the findings of this study are available from the corresponding author upon reasonable request.
